# Assessment of aortic stiffness by cardiovascular magnetic resonance following the treatment of severe aortic stenosis by TAVI and surgical AVR

**DOI:** 10.1186/s12968-016-0256-z

**Published:** 2016-06-10

**Authors:** Tarique Al Musa, Akhlaque Uddin, Timothy A. Fairbairn, Laura E. Dobson, Steven P. Sourbron, Christopher D. Steadman, Manish Motwani, Ananth Kidambi, David P. Ripley, Peter P. Swoboda, Adam K. McDiarmid, Bara Erhayiem, James J. Oliver, Daniel J. Blackman, Sven Plein, Gerald P. McCann, John P. Greenwood

**Affiliations:** Multidisciplinary Cardiovascular Research Centre and The Division of Cardiovascular and Diabetes Research, Leeds Institute for Cardiovascular and Metabolic Medicine, University of Leeds, Leeds, LS2 9JT UK; Division of Medical Physics, Leeds Institute for Cardiovascular and Metabolic Medicine, University of Leeds, Leeds, UK; Department of Cardiovascular Sciences, Cardiovascular Research Centre, Cardiovascular Biomedical Research Unit, University of Leicester, National Institute of Health Research (NIHR), Glenfield General Hospital, Leicester, UK; Leeds Teaching Hospitals NHS Trust, Leeds General Infirmary, Leeds, UK

**Keywords:** Aortic stenosis, Transcatheter aortic valve implantation, Aortic valve replacement, Aortic distensibility, Pulse wave velocity, Cardiovascular magnetic resonance

## Abstract

**Background:**

Aortic stiffness is increasingly used as an independent predictor of adverse cardiovascular outcomes. We sought to compare the impact of transcatheter aortic valve implantation (TAVI) and surgical aortic valve replacement (SAVR) upon aortic vascular function using cardiovascular magnetic resonance (CMR) measurements of aortic distensibility and pulse wave velocity (PWV).

**Methods and results:**

A 1.5 T CMR scan was performed pre-operatively and at 6 m post-intervention in 72 patients (32 TAVI, 40 SAVR; age 76 ± 8 years) with high-risk symptomatic severe aortic stenosis. Distensibility of the ascending and descending thoracic aorta and aortic pulse wave velocity were determined at both time points. TAVI and SAVR patients were comparable for gender, blood pressure and left ventricular ejection fraction. The TAVI group were older (81 ± 6.3 vs. 72.8 ± 7.0 years, *p* < 0.05) with a higher EuroSCORE II (5.7 ± 5.6 vs. 1.5 ± 1.0 %, *p* < 0.05). At 6 m, SAVR was associated with a significant decrease in distensibility of the ascending aorta (1.95 ± 1.15 vs. 1.57 ± 0.68 × 10^−3^mmHg^−1^, *p* = 0.044) and of the descending thoracic aorta (3.05 ± 1.12 vs. 2.66 ± 1.00 × 10^−3^mmHg^−1^, *p* = 0.018), with a significant increase in PWV (6.38 ± 4.47 vs. 11.01 ± 5.75 ms^−1^, *p* = 0.001). Following TAVI, there was no change in distensibility of the ascending aorta (1.96 ± 1.51 vs. 1.72 ± 0.78 × 10^−3^mmHg^−1^, *p* = 0.380), descending thoracic aorta (2.69 ± 1.79 vs. 2.21 ± 0.79 × 10^−3^mmHg^−1^, *p* = 0.181) nor in PWV (8.69 ± 6.76 vs. 10.23 ± 7.88 ms^−1^, *p* = 0.301) at 6 m.

**Conclusions:**

Treatment of symptomatic severe aortic stenosis by SAVR but not TAVI was associated with an increase in aortic stiffness at 6 months. Future work should focus on the prognostic implication of these findings to determine whether improved patient selection and outcomes can be achieved.

## Background

Aortic valve stenosis is the most common valve disease in the western world [1] with a burden that is excepted to double within the next 50 years. With over 50 years of experience, surgical aortic valve replacement (SAVR) is considered the gold standard therapy for symptomatic severe aortic stenosis. However, many patients are considered too high risk for conventional surgery and transcatheter aortic valve implantation (TAVI) is now an established treatment option for those deemed inoperable [2].

Degenerative aortic stenosis can be viewed as part of a continuum that comprises not only valvular dysfunction but also a reduction in aortic compliance [3] which independently contributes to increased afterload [4] and decreased left ventricular function. Increased aortic stiffening is detrimental to arterio-ventricular coupling and coronary perfusion and is an independent predictor of future cardiovascular events and mortality in the general population, essential hypertension, diabetes mellitus, end stage renal failure and in the elderly [5]. Measurement of aortic stiffness is therefore increasingly used in clinical practise as a prognostic indicator.

Cardiovascular magnetic resonance (CMR) offers a robust, reproducible, non-invasive method of assessing both local and regional properties of the aortic wall [6]. Two standard indices of aortic stiffness can be expressed; aortic distensibility and pulse wave velocity, and there is a strong inverse linear relationship reported between these two measurements [7, 8].

The elastic property of the aorta is in part dependent upon the perfusion of the aortic wall via vasa vasorum flow. We hypothesised, based upon the difference in techniques, that more favourable measures of aortic stiffness would be observed following TAVI rather than SAVR. Ultimately, this may herald prognostic implications and guide future patient selection.

The primary aim of this study was to use CMR to serially compare the effects of TAVI and SAVR on aortic stiffness, before and 6 m after treatment for severe symptomatic aortic stenosis.

## Methods

### Study population

A total of 127 patients were prospectively recruited with severe trileaflet degenerative AS after being referred for either TAVI (*n* = 77) or SAVR (*n* = 50) at the University Hospitals of Leeds and Leicester, UK, between July 2008 and December 2013. Severe AS was classified by transthoracic echocardiography (TTE) as an aortic valve area of ≤1.0 cm^2^. Decision for TAVI was taken by a multidisciplinary heart team in accordance with international guidance. Older, higher-risk (higher EuroSCORE) SAVR patients were preferentially recruited wherever possible to facilitate comparable baseline demographics. Exclusion criteria included any contraindication to CMR as well as patients with a known bicuspid aortic valve or aortopathy. The study was approved by the national ethics committee (NRES Committee Yorkshire & the Humber—Leeds West, UK), complied with the Declaration of Helsinki and all patients provided written informed consent.

### Transcatheter aortic valve implantation

TAVI was performed under general anaesthesia by high-volume operators with >5 years’ experience. Either an 18 F CoreValve Revalving system (CVR, Medtronic, Minneapolis, Minnesota, USA) or an 18 F or 20 F Lotus™ Aortic Valve system (Boston Scientific Corporation, Natick, USA) were deployed as previously described [9, 10].

### Surgical aortic valve replacement

SAVR was performed by standard midline sternotomy with cardiopulmonary bypass and mild hypothermia. Biological or mechanical prostheses of varying sizes were used according to surgical preference; concomitant coronary artery bypass grafting was performed as indicated. No patient underwent aortic root or ascending aortic reconstruction.

### CMR protocol

For each individual patient, identical baseline pre-operative and 6-month post-operative scans were performed on the same 1.5 T MR system (Phillips Intera, Best, The Netherlands or Siemens Avanto Erlangen, Germany).

Multi-slice, multi-phase cine imaging was performed using a standard steady-state free precession (SSFP) pulse sequence in the vertical long-axis, four-chamber, left ventricular outflow tract (LVOT) views, and in the short axis (breath-held, 10 mm thickness, 0 mm gap, acquired temporal resolution 30 phases, typical field of view (FOV) 340 mm, acquired spatial resolution 1.88 × 1.88 × 10 mm, TR 3.1 ms, TE 1.55 ms) to cover the entire left and right ventricles. Through-plane velocity encoded (VENC) phase contrast imaging was performed perpendicular to the aortic valve jet at the aortic sinotubular junction (VENC 250–500 cm/s, retrospective gating, slice thickness 6 mm, acquired temporal resolution 40 phases, typical FOV 340 mm, acquired spatial resolution 1.56 × 2.23 × 6 mm, TR 5 ms, TE 3 ms, breath-held, which for a heart rate of 60 beats per minute was equivalent to 13 s).

For aortic distensibility, brachial artery blood pressure was recorded by Dinamap (Critikon, Tampa, USA) immediately prior to high temporal resolution multi-phase SSFP cine imaging (retrospective gating, slice thickness 8 mm, acquired spatial resolution 1.07 × 1.8 × 8 mm, acquired temporal resolution 50 phases, TR 3 ms, TE 1.5 ms, breath-held, which for a heart rate of 60 beats per minute was equivalent to 12 s) acquired transverse to the ascending and descending thoracic aorta at the level of the pulmonary artery bifurcation. Aortic pulse wave velocity was assessed using identical geometric planning with retrospectively gated, through-plane, phase-contrast velocity encoded images (single slice, 8 mm thick, acquired spatial resolution 2.5 × 2.67 × 8 mm, TR 4.6 ms, TE 2.7 ms, acquired temporal resolution 50 phases, typical FOV 350, and VENC 200–500 cm/s, breath-held, which for a heart rate of 60 beats per minute was equivalent to 13 s).

### CMR image analysis

Image analysis was performed in line with international guidance [11], blinded to patient details, using off-line commercially available software (QMass V7.5 and QFlow V7.2, Medis, Leiden, The Netherlands). Standard criteria were employed to delineate LV endocardial and epicardial borders at end-diastole and end-systole for LV mass and volumes. Papillary muscles were excluded from the LV cavity and included within the LV mass for the purpose of analysis. Aortic valve flow indices were quantified using cross-sectional phase contrast images with contouring of the aortic lumen to derive peak forward flow velocity (m/s), and forward and backward flow volumes (ml), for the calculation of trans-valvular pressure gradient and regurgitant fraction (%).

**Fig. 1:**
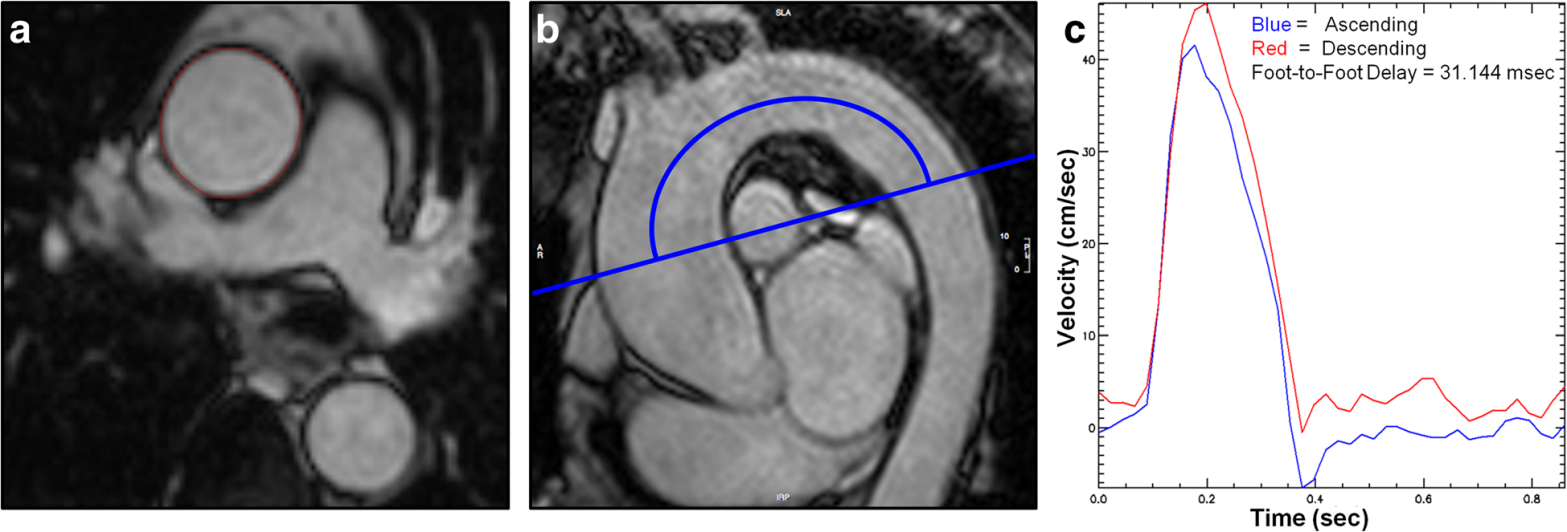
**a** Ascending aortic cross-sectional measurements made by manual planimetry of the aortic endovascular-blood pool interface at minimal and maximal distension. **b** Sagittal oblique CMR image from which the length of the aortic arch is manually measured. The image is subsequently used to determine site of acquisition of phase contrast cines. **c** Time-Velocity curve derived using PMI software to calculate foot-foot delay (curves are automatically adjusted/overlaid to accommodate time delay)

To derive the aortic distensibility of the ascending and descending thoracic aorta, cross sectional measurements were made by manual planimetry of the endovascular-blood pool interface for each phase to determine the maximal and minimal aortic dimensions (Fig. 1a). Aortic distensibility (mmHg^−1^) was calculated using the equation:

$$ \mathrm{Distensibility} = \left(\mathrm{Aortic}\  \max\ \mathrm{lumen}\ \mathrm{area} - \mathrm{Aortic}\  \min\ \mathrm{lumen}\ \mathrm{area}\right)\ /\ \left(\mathrm{Aortic}\  \min\ \mathrm{lumen}\ \mathrm{area}\ \mathrm{x}\ \left[\mathrm{Systolic}\ \mathrm{B}\mathrm{P}\ \hbox{--}\ \mathrm{Diastolic}\ \mathrm{B}\mathrm{P}\right]\right) $$

Aortic PWV (m/s) was calculated by dividing the distance separating two locations and the transit time needed to cover this distance [12]. Analysis was performed using a validated software (PMI v0.4, https://github.com/plaresmedima/PMI-0.4-Runtime-CMRLeeds) based on IDL 6.4 (ITT Visual Information Systems, Boulder, USA) [13]. The distance between the ascending and descending aorta was measured manually from the sagittal/oblique cines of the aortic arch (Fig. 1b). Transit time was calculated using the foot-foot delay method from velocity encoded images of the ascending and descending aorta, manually contoured to derive velocity-time curves (Fig. 1c) [14].

### Statistical analysis

Continuous variables are presented as mean ± SD. Normality was determined by the Shapiro-Wilk test. Frequencies are reported as number (%). The Student *t* test and Wilcoxon signed rank test were used for continuous variables, and *χ*2 or Fisher’s exact test for categorical comparisons. Changes over time were assessed for differences between the treatment groups and clinical variables by two-way repeated measures analysis of variance (ANOVA). Predictors of functional change were evaluated by a stepwise linear regression model with baseline measurements entered as covariate factors. All statistical analyses were performed using PASW software (V.21.0 SPSS, IBM, Chicago, USA); two-sided *p* < 0.05 considered statistically significant.

## Results

**Fig. 2:**
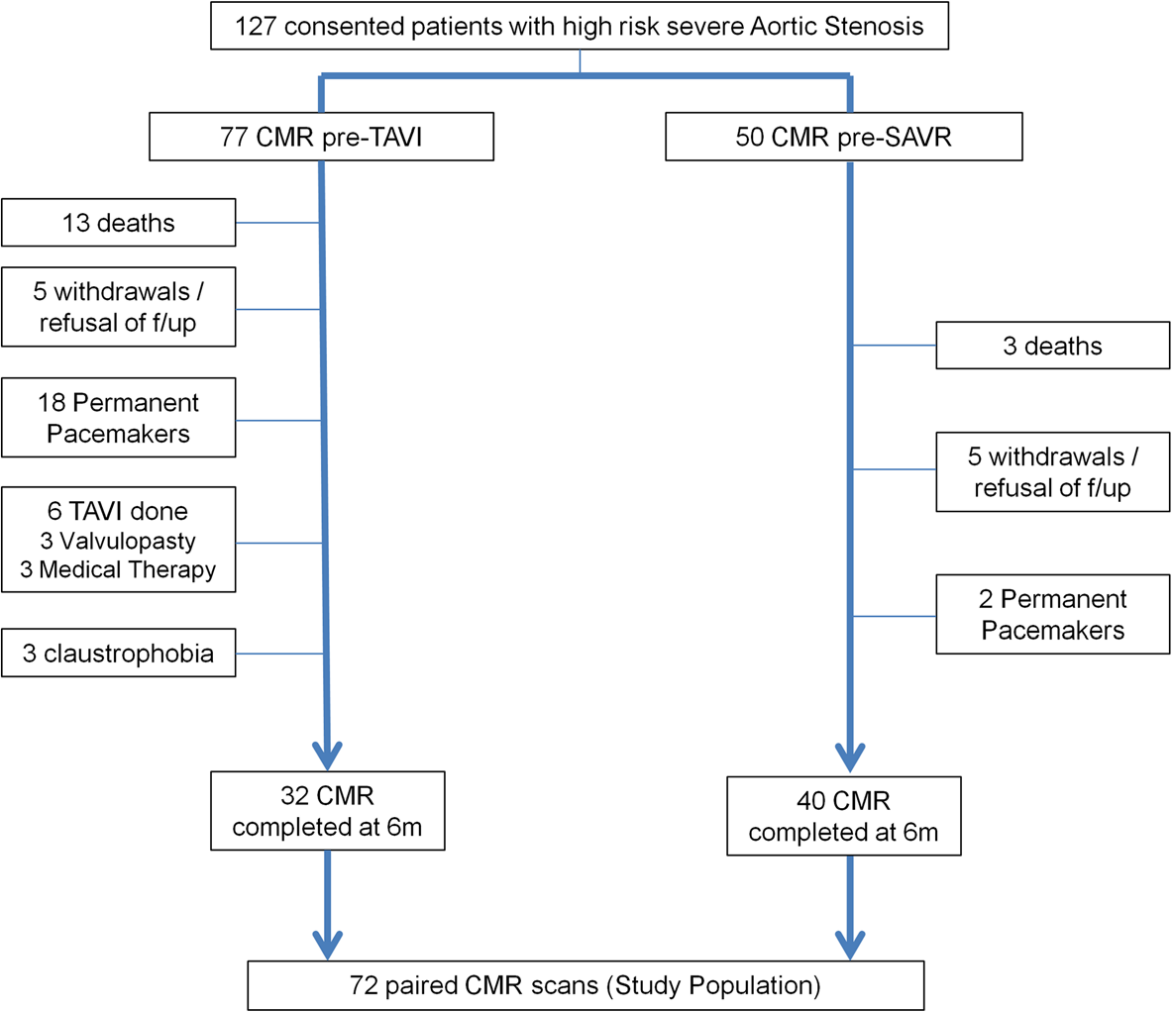
Patient recruitment pathway

Seventy-two patients (32 TAVI, 40 SAVR) with paired pre-operative and 6 m post-operative CMR scans were included for analysis. Reasons for non-completion of the CMR protocol were varied and are depicted in Fig. 2. Baseline characteristics of the study population are shown in Table 1. For both individual groups, key demographic and haemodynamic parameters of the excluded patients were not statistically different to those included for analysis (Table 2), indicating that our study patients were representative of the larger population. The TAVI group were older with a higher predicted 30day mortality risk. The aortic dimensions between the SAVR and TAVI groups were both equivalent and within published normal reference ranges [15] in keeping with our exclusion criteria (Table 1).

**Table 1:** Patient characteristics and baseline data

Characteristics	SAVR (*n* = 40)	TAVI (*n* = 32)	*p* value*
Age	72.8 ± 7.0	81 ± 6.3	0.001
Male gender, *n* (%)	31 (78)	20 (63)	0.151
EuroSCORE II (%)	1.53 ± 1.0	5.66 ± 5.6	0.001
STS Mortality (%)	2.01 ± 0.6	5.68 ± 3.8	0.001
BMI (kgm^−2^)	27.9 ± 6.3	26.6 ± 2.8	0.274
Systolic BP (mmHg)	131 ± 23	127 ± 28	0.696
Diastolic BP (mmHg)	73 ± 11	65 ± 11	0.003
Resting Heart Rate (bpm)	64 ± 12	65 ± 11	0.713
NYHA class	2.5 ± 0.6	3 ± 1.0	0.002
Previous MI, *n* (%)	5 (13)	6 (19)	0.560
Previous PCI, *n* (%)	1 (3)	10 (31)	0.001
Previous CABG, *n* (%)	0 (0)	12 (38)	0.001
Stroke/TIA, *n* (%)	7 (18)	4 (13)	0.667
Peripheral vascular disease, *n* (%)	1 (3)	6 (19)	0.028
Diabetes Mellitus, *n* (%)	4 (10)	8 (25)	0.125
Hyperlipidaemia, *n* (%)	24 (60)	20 (63)	0.977
COPD, *n* (%)	4 (10)	6 (19)	0.358
Atrial Fibrillation, *n* (%)	1 (3)	8 (25)	0.006
eGFR (ml/min/1.73 m^2^)	71.7 ± 13.3	61.4 ± 17.1	0.006
AVA (cm^2^)	0.90 ± 0.5	0.62 ± 0.2	0.002
Mean PG (mmHg)	43 ± 15.8	51 ± 13.7	0.023
LVEF (%)	52 ± 12	52 ± 13	0.961
ValvuloArterial Impedance (Z_va_)	3.88 ± 0.9	4.06 ± 1.6	0.982
Median prosthetic replacement size (mm)	23	27	0.001
Sinuses of Valsalva dimension indexed to BSA (mm/m^2^)	17.9 ± 2.2	18.1 ± 2.1	0.615
Proximal ascending aortic dimension indexed to BSA (mm/m^2^)	17.4 ± 2.8	16.9 ± 2.8	0.505

Values are mean ± SD or *n* (%)

*Abbreviations*: *AVA* aortic valve area, *CABG* coronary artery bypass grafting, *eGFR* estimated glomerular filtration rate, *COPD* chronic obstructive pulmonary disease, *MI* myocardial infarction, *MPG* mean pressure gradient by transthoracic echo, *NYHA* New York Health Association, *PCI* percutaneous coronary intervention, *SAVR* surgical aortic valve replacement, *TAVI* transcatheter aortic valve implantation, *Z*_*va*_ valvuloarterial impedance (systolic arterial pressure + mean echocardiographic transvalvular gradient / stroke volume index), *LVEF* left ventricular ejection fraction, *BSA* body surface area

*p value for comparison between procedure types

**Table 2:** Comparison of baseline demographics and aortic stiffness between included and excluded TAVI and SAVR patients

Parameter	SAVR included	SAVR excluded	*p* value	TAVI included	TAVI excluded	*p* value
Age (years)	72.8 ± 7.0	70.0 ± 8.4	0.261	81 ± 6.3	80 ± 7.2	0.593
STS score (%)	2.01 ± 0.6	2.06 ± 0.9	0.900	5.68 ± 3.8	5.34 ± 2.8	0.693
EuroSCORE II (%)	1.53 ± 1.0	1.35 ± 0.56	0.116	5.66 ± 5.6	5.18 ± 3.4	0.691
Systolic BP (mmHg)	131 ± 23	127 ± 23	0.557	127 ± 28	132 ± 19	0.524
Previous MI (*n* (%))	5 (13)	1 (10)	0.611	6 (19)	4 (15)	0.844
Previous PCI (*n* (%))	1 (3)	2 (20)	0.250	10 (31)	8 (31)	0.872
Peripheral Vascular Disease (*n* (%))	1 (3)	1 (10)	0.531	6 (19)	6 (23)	0.581
Diabetes Mellitus (*n* (%))	4 (10)	4 (40)	0.163	8 (25)	5 (19)	0.721
Hyperlipidaemia, *n* (%)	24 (60)	8 (80)	0.925	20 (63)	15 (58)	0.998
COPD, *n* (%)	4 (10)	1 (10)	0.801	6 (19)	5 (19)	0.849
Atrial Fibrillation, *n* (%)	1 (3)	3 (10)	0.121	8 (25)	5 (19)	0.721
AVA (cm^2^)	0.90 ± 0.5	0.67 ± 0.2	0.195	0.62 ± 0.2	0.62 ± 0.2	0.941
AAD (×10^−3^mmHg^−1^)	1.95 ± 1.15	2.12 ± 1.07	0.648	1.96 ± 1.51	1.55 ± 0.61	0.338

*Abbreviations*: *STS* Society of Thoracic Surgeons mortality risk score, *AVA* aortic valve area, *AAD* ascending aortic distensibility

### Procedural data

For the TAVI group, 25(78 %) patients received a Medtronic CoreValve and 7(22 %) a Boston Scientific Lotus valve. The femoral access route was used for 30(94 %) and the subclavian artery for the remaining 2(6 %) patients. Procedural success was 100 % with an average catheterisation time of 159 ± 48 min, fluoroscopy time of 25 ± 7 min and 146 ± 48 ml of contrast administered.

In the surgical group, five patients received a mechanical prosthesis (either Sorin Carbomedics or St Jude’s mechanical) and the remaining 35(88 %) a tissue bioprosthesis (Sorin mitroflow, Edwards Perimount Magna, Medtronic Hancock, Hancock II and Mosaic, Vascutek Terumo Aspire). Eleven (28 %) received concomitant coronary bypass grafting, of which 6 involved use of the left internal mammary artery. For the group as a whole, the average bypass time was 108 ± 50 min and average cross clamp time 81 ± 43 min. The average length of stay in intensive care was 3.5 ± 2.8 days.

### Aortic valve haemodynamics and LV reverse remodelling

Results of the baseline and 6 m CMR scans are shown in Table 3. No significant change in arterial pulse pressure was observed following SAVR (58.8 ± 18.6 vs. 61.4 ± 14.4 mmHg, *p* = 0.402) or TAVI (63.5 ± 24.0 vs. 69.7 ± 20.3 mmHg, *p* = 0.203). There was no significant change in the number of antihypertensive medications used, neither following SAVR (1.3 ± 0.8 vs. 1.4 ± 0.8, *p* = 0.503) or TAVI (1.2 ± 1.0 vs. 1.3 ± 1.0, *p* = 0.161). Reductions in aortic valve pressure gradient, valvuloarterial impedance, LV mass index and end-diastolic volume index were seen 6 m following both SAVR and TAVI (Table 3).

**Table 3:** Preoperative baseline and 6 month follow-up measurements

	SAVR		TAVI	
	Baseline	6 months	Baseline	6 months
Haemodynamics				
Heart Rate (bpm)	64 ± 12	65 ± 11	65 ± 11	66 ± 15
Systolic BP (mmHg)	131 ± 23	133 ± 20	127 ± 28	134 ± 22
Number of Medications^a^	1.3 ± 0.8	1.4 ± 0.8	1.2 ± 1.0	1.3 ± 1.0
Systemic Arterial Compliance^b^	0.88 ± 0.3	0.74 ± 0.2*	0.81 ± 0.3	0.71 ± 0.2
Aortic Valve				
Peak gradient (mmHg)^c^	59 ± 20	32 ± 18***	54 ± 14	25 ± 13***
Z_va_	3.9 ± 0.9	3.5 ± 0.8*	4.1 ± 1.6	3.0 ± 1.1**
Left Ventricle				
Mass Index (g/m^2^)	80 ± 25	65 ± 16***	82 ± 22	68 ± 18***
EDVI (ml/m^2^)	95 ± 25	77 ± 14***	95 ± 25	86 ± 19*
EF (%)	52 ± 12	57 ± 8**	52 ± 13	55 ± 11

*Abbreviations*: *EDVI* end diastolic volume indexed to body surface area, *EF* ejection fraction, *Zva* valvuloarterial impedance

Paired *t* test to compare baseline and 6 months: *p < 0.05, **p < 0.01, ***p < 0.001

^a^defined as any of: ACE inhibitor, angiotensin II receptor antagonist, β blocker, spironolactone, doxazosin, hydralazine, amlodipine, felodipine or bendrofluazide

^b^Derived as stroke volume index / pulse pressure

^c^Derived from CMR assessment

### Aortic stiffness indices

**Fig. 3:**
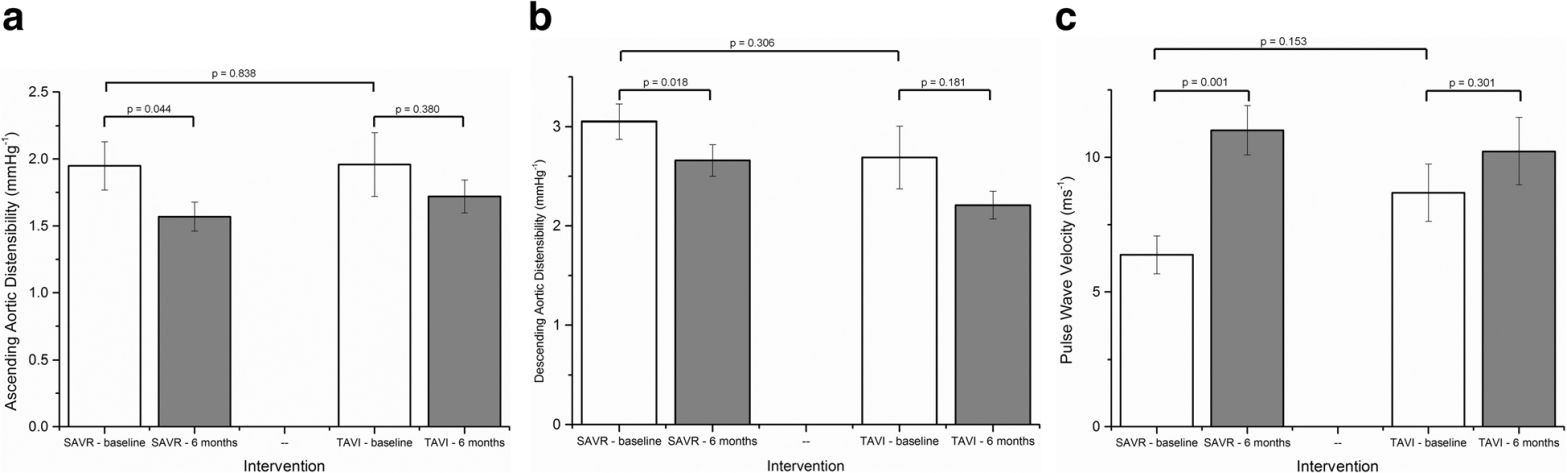
Bar charts depicting change in indices of aortic stiffness (3 (**a**) ascending aortic distensibility, 3 (**b**) descending aortic distensibility, 3 (**c**) pulse wave velocity) seen pre- and post-SAVR and TAVI (mean and standard error bars)

At baseline there was no difference between the groups in respect to PWV (*p* = 0.153) or distensibility; neither of the ascending (*p* = 0.838) or descending thoracic aortic (*p* = 0.306). Change in indices of aortic stiffness are shown in Fig. 3.

At 6 m, SAVR was associated with a decrease in distensibility of the ascending aorta (1.95 ± 1.15 vs. 1.57 ± 0.68 × 10^−3^mmHg^−1^, *p* = 0.044) and of the descending thoracic aorta (3.05 ± 1.12 vs. 2.66 ± 1.00 × 10^−3^mmHg^−1^, *p* = 0.018). There was a concomitant increase in PWV observed at 6 m (6.38 ± 4.47 vs. 11.01 ± 5.75 ms^−1^, *p* = 0.001) (Table 4). These changes were independent of whether or not bypass grafting occurred at the time of valve replacement.

**Table 4:** Change in mean measurements pre- and post SAVR and TAVI

	SAVR			TAVI		
	Pre	Post	*p* value*	Pre	Post	*p* value*
Ascending AD (×10^−3^mmHg^−1^)	1.95 ± 1.15	1.57 ± 0.68	0.044	1.96 ± 1.51	1.72 ± 0.78	0.380
Descending AD (×10^−3^mmHg^−1^)	3.05 ± 1.12	2.66 ± 1.00	0.018	2.69 ± 1.79	2.21 ± 0.79	0.181
Aortic Arch PWV (m/s)	6.38 ± 4.47	11.01 ± 5.75	0.001	8.69 ± 6.76	10.23 ± 7.88	0.301
Change in AA area** (mm^2^)	99 ± 54	80 ± 42	0.032	85 ± 32	91 ± 38	0.410
Change in DA area*** (mm^2^)	87 ± 26	80 ± 27	0.083	73 ± 28	74 ± 25	0.916
Pulse pressure (mmHg)	58 ± 19	61 ± 14	0.322	63 ± 24	70 ± 20	0.150
Length of aortic arch (mm)	139 ± 18	134 ± 20	0.129	126 ± 21	122 ± 16	0.223

*paired samples *t*-test

**defined as maximal– baseline cross-sectional ascending aortic area

***defined as maximal– baseline cross-sectional descending aortic area

**Fig. 4:**
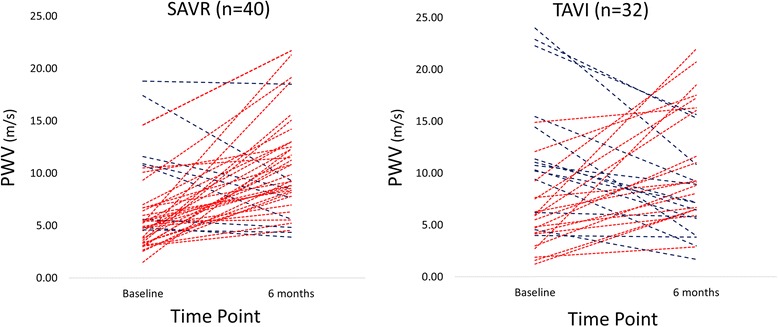
Changes in PWV between baseline and 6 months following TAVI and SAVR. (red lines indicate an increase, blue lines represent a decline)

There was no significant change observed in either the distensibility of the ascending aorta (1.96 ± 1.51 vs. 1.72 ± 0.78 × 10^−3^mmHg^−1^, *p* = 0.380) or of the descending thoracic aorta (2.69 ± 1.79 vs. 2.21 ± 0.79 × 10^−3^mmHg^−1^, *p* = 0.181) following TAVI. Similarly, TAVI was not associated with any significant change in PWV at 6 m (8.69 ± 6.76 vs. 10.23 ± 7.88 ms^−1^, *p* = 0.301) (Fig. 4).

### Demographic and procedural factors associated with change in aortic stiffness

In linear regression analysis, no baseline characteristic (including age, gender, eGFR, surgical risk score or Zva) or procedural variable (including surgical times, CABG and valve type or size) was found to be associated with any index of increased aortic stiffness after SAVR.

## Discussion

Aortic physiology is central to governing the entire cardiovascular network, serving as a conduit and also regulating coronary perfusion and LV performance. Only a limited number of studies have evaluated aortic vascular function following intervention for severe aortic stenosis [16–21]. The use of M-mode transthoracic echocardiography to determine proximal aortic distensibility has been the exclusive method of investigation; none have measured local and regional indices simultaneously and none have sought to compare SAVR directly with TAVI.

Using CMR we have been able to demonstrate that treatment of severe aortic stenosis with SAVR, compared to TAVI, is associated with an increase in aortic stiffness at 6 months independent of baseline characteristics. This was consistently defined by non-invasive measurement of both local indices (ascending and descending thoracic aortic distensibility) and a regional index (aortic arch pulse wave velocity).

The elastic properties of the aorta relate to its inherent histological structure, the influence of the autonomic nervous system and the perfusion of the aortic wall via vasa vasorum flow [16]. In this regard, the fundamental difference in the techniques of SAVR and TAVI could explain our observations.

SAVR involves aortotomy and traumatises aortic wall integrity with destruction of the vasa vasorum. The removal the periaortic fat (containing the vasa vasorum) from the ascending aorta in animal studies has been shown to worsen aortic distensibility acutely due to ischemic medial necrosis and altered fibre composition [22]. In a porcine model, histological analysis of avascular aorta following surgical manipulation revealed abnormal straightening of the elastin and collagen fibers of the outer media, resulting in increased aortic stiffness under a wide range of stresses [23] .

A previous study of 31 patients (mean age 67.2 years) found a significant reduction in ascending aortic distensibility at 7 days following mechanical AVR (from 2.21 to 1.01), with a recovery towards pre-operative levels at 6 months (1.79) [19]. The authors suggested that the aetiology was due to “aortic root stunning” implicating surgical trauma to the aortic wall via cannulation, cooling, clamping, incising and then suturing, all of which disrupts the aortic wall continuity. None of this occurs during a conventional TAVI procedure and this may underscore the findings of our study.

Our study may have missed the early period of aortic root stunning as we did not examine aortic stiffness acutely and thus cannot comment on temporal trends post SAVR. However our study does suggest that the significant increase in aortic stiffness persists for at least at 6 months. A decrease in distensibility of the descending thoracic aorta has not previously been reported. This finding suggests the surgical insult affects the aorta more globally, extending beyond the point of local clamp contact.

Interestingly, we found that bypass time, cross clamp time, valve size, valve type and concomitant coronary bypass grafting were not associated with a decline in any of the parameters of aortic stiffness. This suggests the deterioration in aortic stiffness seen at the 6 m time point is insensitive to modifiable surgical technique.

Progressive fragmentation of aortic elastin occurs throughout adulthood and underlies a reduction in the Windkessel effect of the aorta, elevating pulse pressures for a given stroke volume [24]. A recent CMR study measuring both AD and PWV study in healthy subjects reported that aortic segments stiffen with age, but that after the age of 57 years, the ascending aorta is stiffer than the descending thoracic aorta [25]. We also observed greater distensibility in the descending thoracic aorta compared to the ascending aorta in both groups at baseline which reflects this physiological process.

Measures of aortic stiffness, and PWV in particular, exhibit a strong dependence upon age [26] which must be factored into the interpretation of our findings. We have studied two groups with an age difference of approximately 9 years; a reflection of current TAVI implantation criteria. Although the two groups were statistically comparable for PWV at baseline, in absolute terms the PWV values are dissimilar (Fig. 3c); caution is thus required in any clinical interpretation of change in PWV in these two populations. However, the ascending AD of the SAVR group and TAVI group were very similar (1.95 vs. 1.96), yet at 6 months, a statistically significant decline was seen in the SAVR group, but not following TAVI.

It is noteworthy the deleterious effect seen following SAVR was independent of age when entered as a statistical covariate, challenging its potential use in patient selection pre-operatively. Whilst an ascending AD and PWV of 1.72 × 10^−3^mmHg^−1^ and 10.23 m/s may be acceptable and expected in patients aged 80 (post-TAVR), worse values of 1.57 × 10^−3^mmHg^−1^ and 11.01 m/s may not necessarily be acceptable in patients 9 years younger (post-SAVR), undergoing an intervention for prognostic reasons who inherently have a lower surgical risk score.

We and others have shown that lowering of blood pressure can improve aortic stiffness [5, 27, 28]. However in this study, blood pressure and pharmacotherapy were unchanged pre- and post-procedure, suggesting that this was unlikely to account for the difference in impact upon aortic stiffness between TAVI and SAVR. The normal systemic arterial compliance in both groups indicates that baseline haemodynamics were governed predominantly by aortic valve disease without any associated aortic or LV pathology [4]. Following intervention, SAVR was associated with a limited decrease in Z_va_ as opposed to TAVI. Given a comparable and important reduction in aortic valve gradient (valvular load), the dampened Z_va_ response to SAVR likely reflects an increase in arterial load reflecting a mechanical deterioration in aortic function.

The effect of TAVI upon proximal aortic distensibility has been assessed once previously in 30 patients (mean age 79.9 years) using echocardiography 7 days post-procedure [16]. No significant change was observed with an AD of 1.89 pre and 2.05 post TAVI. Our study supports these findings and additionally demonstrates preservation of local and regional aortic stiffness at 6 months post-TAVI. Our study indicates the absence of deterioration in aortic stiffness out to 6 months post-TAVI may favour its usage over SAVR in younger patient populations.

The motivation for this study was to investigate whether TAVI or SAVR is more favourable upon aortic stiffness and thus potentially prognosis. It might be expected that our findings would translate into an increased incidence of adverse cardiovascular events in the surgical population. From a meta-analysis of 17 longitudinal studies comprising 15,877 subjects, an increase in aortic PWV by 1 m/s corresponded to an age-, sex-, and risk factor-adjusted risk increase of 15 % in all-cause mortality [29]. A dramatic increase in PWV was seen following SAVR in our study although our follow-up data of the surgical group extends to an average of 2.8 years with 95 % (*n* = 38) of subjects surviving, such that the numbers are insufficient to make any direct inference.

In the US CoreValve High Risk Study, a higher survival rate at 1 year in patients undergoing TAVI compared directly with SAVR was likely due to more rapid recovery coupled with relatively lower rates of stroke [2]. Our findings are noteworthy in this respect as aortic stiffness may be a contributory factor to this observation. Indeed, in a study of 310 patients aged 50 years or more, lower aortic distensibility was shown to be an independent predictor of all-cause mortality in patients presenting with first-ever acute ischemic stroke [30]. Larger studies with longer follow-up post AVR are required to determine the precise predictive power of aortic stiffness with respect to mortality and morbidity in this setting.

### Study limitations

The main limitation is the attrition of patients who were unable to complete the CMR protocol at 6 months. This was predominantly in the TAVI population who are a very challenging group to study due to age, frailty and comorbidity. Mortality and pacemaker rates were high, but consistent with large international registries. A small number of TAVI patients declined follow up because of deteriorating health and transfer into long-term nursing care. This is one of the largest studies of its kind and patients not studied at 6 m were not statistically different in demographics to those that were. Nonetheless, the potential for bias cannot be excluded as the sickest patients who withdrew may have had higher post-procedural arterial stiffness and worst outcomes. Furthermore, our final analysed patient groups were relatively small, giving limited power to report ‘no difference’ in baseline variables such as PWV, thus raising the possibility of Type 1 and Type 2 errors, and transfer bias influencing our final group comparisons. It is possible that our TAVI population, with an average age of 80 years, may have reached near maximal aortic stiffness. Thus TAVI itself may be deleterious or even beneficial to aortic stiffness, but our particular population we have not been able to elucidate this.

The difference in baseline demographics between the groups was unavoidable due to current TAVI implantation criteria. However, our study remains unprecedented, and can be considered a real life reflection, at least of UK national practice [31]. Finally, this study has not assessed patients undergoing isolated on-pump coronary bypass surgery or direct aortic TAVI.

## Conclusion

In this two centre comparative study using CMR-derived measurements, treatment of symptomatic severe aortic stenosis by SAVR but not TAVI was associated with an increase in aortic stiffness from baseline to 6 m. Given aortic stiffness is a marker of adverse cardiovascular events, future work should focus on the potential prognostic benefit of TAVI over SAVR, particularly in younger age-matched populations, as TAVI implantation criteria evolve.

### Abbreviations

AAD, ascending aortic distensibility; AD, aortic distensibility; ANOVA, analysis of variance; AVA, aortic valve area; BMI, body mass index; BP, blood pressure; BSA, body surface area; CABG, coronary artery bypass grafting; CMR, cardiovascular magnetic resonance; COPD, chronic obstructive pulmonary disease; EDVI, end diastolic volume index; eGFR, estimated glomerular filtration rate; FOV, field of view; LV, left ventricle; LVOT, left ventricular outflow tract; MPG, mean pressure gradient; NYHA, New York Heart Association; PCI, percutaneous coronary intervention; PWV, pulse wave velocity; SAVR, surgical aortic valve replacement; SD, standard deviation; SSFP, steady state free precession; STS, Society of Thoracic Surgeons; TAVI, Transcatheter aortic valve implantation; TE, echo time; TR, repetition time; VENC, velocity encoded; Zva, valvuloarterial impedance.
